# Ox-LDL Aggravates the Oxidative Stress and Inflammatory Responses of THP-1 Macrophages by Reducing the Inhibition Effect of miR-491-5p on MMP-9

**DOI:** 10.3389/fcvm.2021.697236

**Published:** 2021-10-01

**Authors:** Yiling Liao, Enzheng Zhu, Wanxing Zhou

**Affiliations:** Department of Internal Cardiology Medicine, The First Affiliated Hospital/School of Clinical Medicine of Guangdong Pharmaceutical University, Guangzhou, China

**Keywords:** ox-LDL, miR-491-5P/MMP-9 axis, THP-1 macrophages, oxidative stress, inflammatory response

## Abstract

**Background:** Oxidized low-density lipoprotein (ox-LDL) can induce oxidative stress and inflammatory responses in macrophages to facilitate the genesis and development of atherosclerosis. However, the intermediate links remain unclear. MiR-491-5P can inhibit matrix metalloproteinase 9 (MMP-9); however, it remains unclear whether ox-LDL enhances MMP-9 expression and aggravates the oxidative stress and inflammatory responses under the mediating effect of miR-491-5P.

**Method:** THP-1 macrophages were divided into 10 groups: blank (control), model (ox-LDL), miR-491-5P high-expression (miR-491-5P mimic), miR-491-5P control (mimic-NC), MMP-9 high-expression (MMP-9-plasmid), MMP-9 control (plasmid-NC), miR-491-5P+plasmid-NC, miR-491-5P+ MMP-9-plasmid, MMP-9 gene silencing (MMP-9-siRNA), and gene silencing control (siRNA-NC). The cells were transfected for 48 h and then treated with 50 μg/mL of ox-LDL for 24 h. MMP-9 mRNA and miR-491-5P expression levels in the cells were detected using reverse transcription-quantitative polymerase chain reaction, and the MMP-9 levels were detected with western blotting. The levels of oxidative stress factors (malondialdehyde [MDA]), reactive oxygen species (ROS), and antioxidant factors (superoxide dismutase [SOD]), and the expression levels of inflammatory factors (tumor necrosis factor [TNF-α] and interleukin-1β and−6 [IL-1β and IL-6]) in the supernatant were detected with enzyme-linked immunosorbent assay.

**Results:** MDA, ROS, TNF-α, IL-1β, IL-6, and MMP-9 levels were increased, SOD activity was reduced, and miR-491-5P expression was downregulated in the ox-LDL group compared to the control group. In the miR-491-5P mimic group, the MDA, ROS, TNF-α, IL-1β, IL-6, MMP-9 mRNA and protein levels were downregulated, and SOD activity was enhanced compared to the ox-LDL group. MMP-9-plasmid elevated the MDA, ROS, TNF-α, IL-1β, IL-6, MMP-9 mRNA and protein levels, and downregulated SOD activity and miR-491-5P expression. Following transfection with MMP-9-siRNA, the MMP-9-plasmid outcomes were nullified, and the resulting trends were similar to the miR-491-5p simulation group. Oxidative stress and inflammatory responses were higher in the miR-491-5P mimic+MMP-9-plasmid co-transfection group than in the miR-491-5P mimic group.

**Conclusion:** Ox-LDL aggravates the oxidative stress and inflammatory responses of THP-1 macrophages by reducing the inhibition effect of miR-491-5p on MMP-9.

## Introduction

Oxidative stress and the inflammatory response play critical roles in the development of atherosclerosis (AS) (1). Low-density lipoprotein (LDL) is the primary lipoprotein involved in cholesterol-induced AS. Oxidized LDL (ox-LDL) is extremely pro-inflammatory and has pro-oxidative stress effects (2, 3). Macrophages, derived from mononuclear cells (1), can phagocytize ox-LDL to form lipid-laden foam cells, induce the generation of reactive oxygen species (ROS), secrete pro-inflammatory factors, and cause inflammation of vascular walls and their subsequent repair, leading to the genesis and development of plaques (4, 5). Matrix metalloproteinase 9 (MMP-9) is a proteolytic enzyme that can degrade the extracellular matrix (ECM) in plaque fibrous caps, weakening them and causing plaque instability (6, 7). MMP-9 is present at high expression levels in unstable plaques (8). MMP-9 exerts a positive feedback effect on many pro-inflammatory factors (9) and can aggravate the inflammatory response (10, 11), leading to the development and instability of plaques (12). Ox-LDL can induce macrophages to secrete MMP-9 (13). However, the complete bio-molecular mechanism is not comprehensively understood. MiR-491-5P is a micro-RNA (miRNA), a micromolecular non-coding RNA with a length of 20–22 nucleotides (14). It can inhibit the expression of target mRNA by binding to it, thus exerting a regulatory effect (15, 16). MiR-491-5P achieves targeted inhibition of MMP-9 expression (17). When the regulatory effect of miR-491-5P on MMP-9 is removed, MMP-9 expression is upregulated, thus increasing the incidence of atherosclerotic cerebral infarction (18). However, whether ox-LDL enhances MMP-9 expression and aggravates the oxidative stress and inflammatory responses under the mediating effect of miR-491-5P remains unclear. To address this gap in understanding, *in vitro* cell experiments were conducted and are described here.

## Materials and Methods

### Cell Culture and Grouping

Human monocytes (THP-1) were purchased from the Cell Bank of the Chinese Academy of Sciences. The cells were cultured in RPMI-1640 culture medium (Gibco; Thermo Fisher Scientific, Inc.) supplemented with 10% FBS (Gibco; Thermo Fisher Scientific, Inc.). The F5 cells were incubated in a complete medium containing 100 ng/mL phorbol-12-phorbol myristate acetate (Sigma-Aldrich; Merck KGaA) for 48 h and differentiated into THP-1 macrophages as the experimental cells.

The experimental cells were divided into 10 groups: control, model (ox-LDL), miR-491-5P high-expression (miR-491-5P mimic), miR-491-5P no-load (mimic-NC), MMP-9 high-expression (MMP-9-plasmid), MMP-9 no-load (plasmid-NC), miR-491-5P+plasmid-NC, miR-491-5P+ MMP-9-plasmid, MMP-9 gene silencing (MMP-9-siRNA), and gene silencing control (siRNA-NC). Six wells were used in each group.

### Cell Transfection

The oligonucleotide sequences (Gene Pharma, Shanghai, China) of miR-491-5P mimic (miR-491-5P imported) and miR-491-5P mimic-NC (miR-491-5P control), MMP-9 gene silencing (MMP-9-siRNA), gene silencing control (siRNA-NC), MMP-9 high-expression (MMP-9-plasmid), and plasmid control (plasma-NC) sequences (Santa Cruz Biotechnology) were used to establish the corresponding transfection systems. In each group, lipofectamine^™^ 2000 transfection reagent (Invitrogen Life Technologies, CA, USA) was used to transfect the THP-1 macrophages for 48 h.

### Co-Incubation of Ox-LDL and Transfected Macrophages

The differentiated THP-1 macrophages were incubated using 10, 30, 50, or 70 μg/mL ox-LDL (Sigma, USA) in an incubator (temperature: 37°C, saturated humidity: 5% CO_2_) for 24 h to determine the appropriate ox-LDL concentration (19). The transfected THP-1 macrophages were then treated with 50 μg/mL of ox-LDL (Sigma) for 24 h.

### Analysis of Cell Viability

The cell counting kit-8 (CCK-8) assay was used to analyze cell viability (Abcam, USA). Briefly, THP-1 cells were plated in 96-well plates and differentiated into macrophages. After exposure to 50 μg/mL ox-LDL and cell transfection, 10 μL of CCK-8 working solution was added to each well, and incubation was continued for 4 h at 37°C. The plates were detected at 450 nm using a spectrophotometer (Thermo Fisher Scientific).

### Verification of THP-1 Macrophage Foaming

#### Oil Red O Staining

THP-1 macrophages were incubated with or without ox-LDL and transfected with miR-491-5P mimic, MMP-9-plasmid, MMP-9-siRNA, or miR-491-5P mimic+MMP-9-plasmid for 48 h before exposure to 50 μg/mL ox-LDL. After washing three times with phosphate-buffered saline (PBS), the cells were fixed with 4% paraformaldehyde for 25 min. Oil Red O solution (for cultured cells) was added and the cells were incubated for 30 min at 37°C and counterstained with Mayer Hematoxylin solution, according to the manufacturer's protocol (G1262 Solarbio Beijing, China). After extensive washing with PBS, the cells were immediately photographed using a microscope (Olympus). After removing the staining solution, the dye retained in the cells was eluted into isopropanol and the IOD value of the Oil Red O staining was detected at 540 nm using a spectrophotometer (Thermo Fisher Scientific).

#### Lipid Accumulation Assay

Lipid drop (LD) accumulation was evaluated using BODIPY 493/503 (10 μg/mL; Thermo Fisher, D2148) staining. The nuclei were stained with 4′,6-diamidino-2-phenylindole (DAPI) staining solution. The average LD intensity from BODIPY 493/503 staining was quantified using Image J software (National Institutes of Health).

### Reverse Transcription-Quantitative Polymerase Chain Reaction (RT-qPCR)

Total RNA was extracted from the cells using TRIzol^®^ reagent (Invitrogen; Thermo Fisher Scientific, Inc.). Hi Script TM II Q RT Super Mix (Vazyme Biotech Co., Ltd.) was used for the reverse transcription of total RNA into cDNA. Subsequently, a SYBR Green PCR kit (Vazyme Biotech Co., Ltd.) was used for qPCR. Quantification was undertaken using the 2-ΔΔCq method (20), and the internal references were GAPDH (for mRNA) and U6 (for miRNA). The assays were repeated three times for each sample and the mean value was used. The PCR primer sequences were as follows:

MiR-491-5P forward 5′-GGAGT GGGG AACCCTTCC-3′;
                     reverse 5′-GTGGGGGAGGGGATTC-3′
U6 forward 5′-GCTTC GGAGCACATACT AAAA T-3′;
        reverse 5′-CGCTTCACGAATTT GCGTGTCAT-3′
MMP-9 forward 5′-AGACCTGCGAGGAGATTCCAA3′;
                reverse 5′-CGGAGGAGTCGAGT-3′;
GAPDH forward 5′-CTTGGGGGTA GGAAGGACTC-3′;
                reverse 5′-GTA GAGAGGAGATGATGTTCT-3′.

### Western Blotting Analysis

The cells were pyrolyzed using RIPA pyrolytic buffer solution (Beyotime Institute of Biotechnology, Shanghai, China), and the total protein was extracted. A BCA Protein Assay kit (Beyotime Institute of Biotechnology, Shanghai, China) was used to quantify the total protein. The equivalent amount of protein was treated for 40 min with 12% SDS-PAGE Gel SuperQuick Preparation Kit for electrophoretic separation. The separated protein was transferred to polyvinylidene difluoride membranes (Millipore Corporation) and sealed using 5% skim milk to prevent nonspecific binding. Next, the membranes were incubated with the indicated primary antibodies, including MMP-9 (1:1000, Abcam), and left overnight at 4°C. The primary antibodies were rinsed, and the membrane was incubated with secondary antibodies for 2 h; anti-GAPDH antibody (1:1000, Abcam) was used as the internal reference. After rinsing, the protein bands were assessed using an enhanced chemiluminescence reagent (WBKLS0100, Millipore, USA).

### Detection of Oxidative Stress-Related Factors

A superoxide dismutase (SOD) assay kit (Cayman Chemical Company) was used to determine SOD activity, as per the manufacturer's protocol. Malondialdehyde (MDA) activity was measured using an MDA assay kit (Cayman Chemical Company), according to the manufacturer's protocol. ROS levels were analyzed using a specific kit.

### Enzyme-Linked Immunosorbent Assay (ELISA)

The concentrations of inflammatory cytokines, including tumor necrosis factor α (TNF-α), interleukin-1β (IL-1β), and IL-6, were detected using ELISA kits (R & D, USA). Specific ELISA kits were used to detect the related markers, according to the manufacturer's protocols.

### Statistical Analysis

Statistical analysis was performed using SPSS 26.0, followed by graphical analysis using GraphPad Prism 8.0 software (GraphPad Software Company). The normal distribution test and homogeneity test of variance were undertaken initially, and the normally distributed data were expressed as the mean ± standard deviation (SD). The least significant difference test was used to compare every two groups for homogeneity of variance. One-way analysis of variance was undertaken for multi-group comparisons; non-parametric tests were used in the case of heterogeneity of variance. *P* < 0.05 was interpreted as a statistically significant difference.

## Results

### Ox-LDL Downregulated miR-491-5P in THP-1 Macrophages

**Figure 1:**
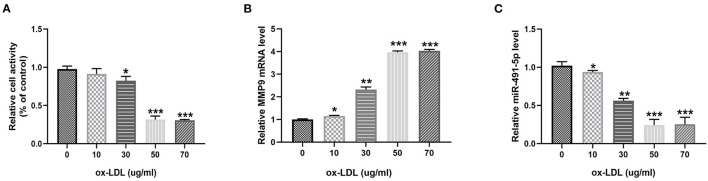
Ox-LDL downregulated miR-491-5P and upregulated MMP-9 in THP-1 macrophages. THP-1 macrophages were induced by different concentrations (0, 10, 30, 50, and 70 μg/mL) of ox-LDL. **(A)** Cell viability was determined using CCK-8. **(B,C)** qRT-PCR analysis measured MMP-9 level and mir-491-5p level in THP-1 macrophages stimulated with different doses of ox-LDL. Data are presented as the mean ± SD, *n* = 6. ^*^P < 0.05, ^**^P < 0.01, and ^***^P < 0.001.

To investigate the effect of ox-LDL on THP-1 macrophages, THP-1 macrophages were treated with ox-LDL at different concentrations (0, 10, 30, 50, and 70 μg/mL) for 24 h. Ox-LDL significantly reduced THP-1 macrophage activity and mir-491-5p expression and upregulated MMP-9 levels in a dose-dependent manner (Figures 1A–C). There was no significant difference between the 50 μg/mL and 70 μg/mL ox-LDL treatments; therefore, the 50 μg/mL concentration was selected for subsequent experiments.

### Ox-LDL Induced Oxidative Stress and Inflammatory Response of THP-1 Macrophages by Regulating mir-491-5p and MMP-9

**Figure 2:**
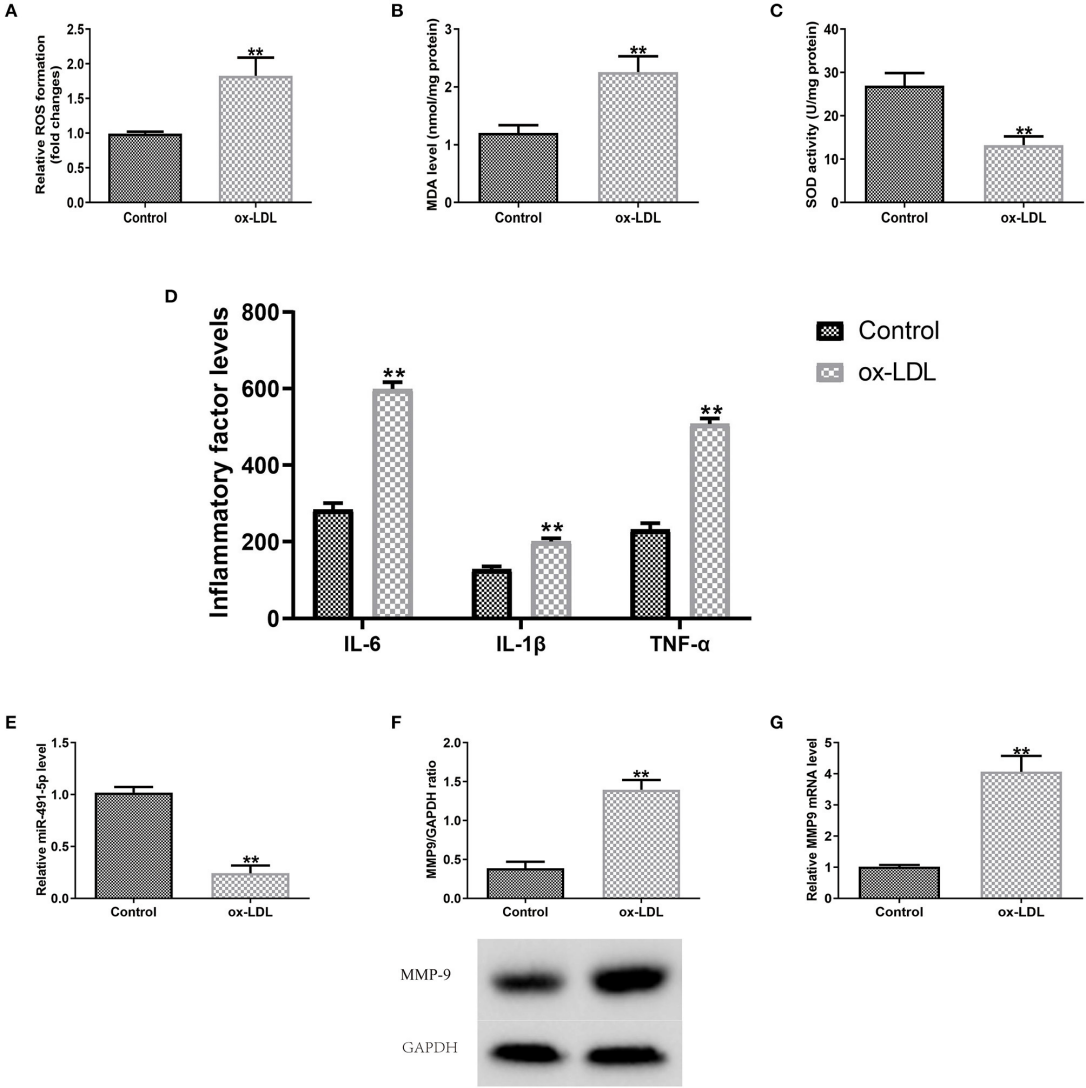
Outcomes of ox-LDL on macrophage expression levels (miR-491-5P and MMP-9), oxidative stress and inflammatory factors. **(A–C)** ROS and MDA levels and SOD activity were measured using commercial kits. **(D)** Levels of inflammatory factors (TNF-α, IL-1β, and IL-6) were detected via ELISA. **(E–G)** MiR-491-5P, MMP-9 protein, and mRNA levels were measured with qRT-PCR, respectively. Data are presented as the mean ± SD, *n* = 6. Compared with the control group, ^**^P < 0.01.

Subsequently, the expression levels of MMP9 and miR-491-5p in ox-LDL-induced THP-1 macrophages oxidative stress and inflammatory response were detected. The ox-LDL group had significantly higher ROS and MDA levels and lower SOD activity than the control group (Figures 2A–C). ELISA results showed that the ox-LDL group increased the secretory levels of TNF-α, IL-1β, and IL-6 (Figure 2D). RT-qPCR/western blotting analyses showed that ox-LDL induced downregulation of miR-491-5p expression (Figure 2E) and upregulation of MMP-9 protein (Figure 2F) and mRNA (Figure 2G) expression in THP-1 macrophages. These results indicated that mir-491-5P and MMP-9 might mediate oxidative stress and inflammatory response induced by ox-LDL.

### MMP-9 Overexpression and Knockdown Promoted and Inhibited Ox-LDL-Induced Oxidative Stress and Inflammatory Response in THP-1 Macrophages, Respectively

**Figure 3:**
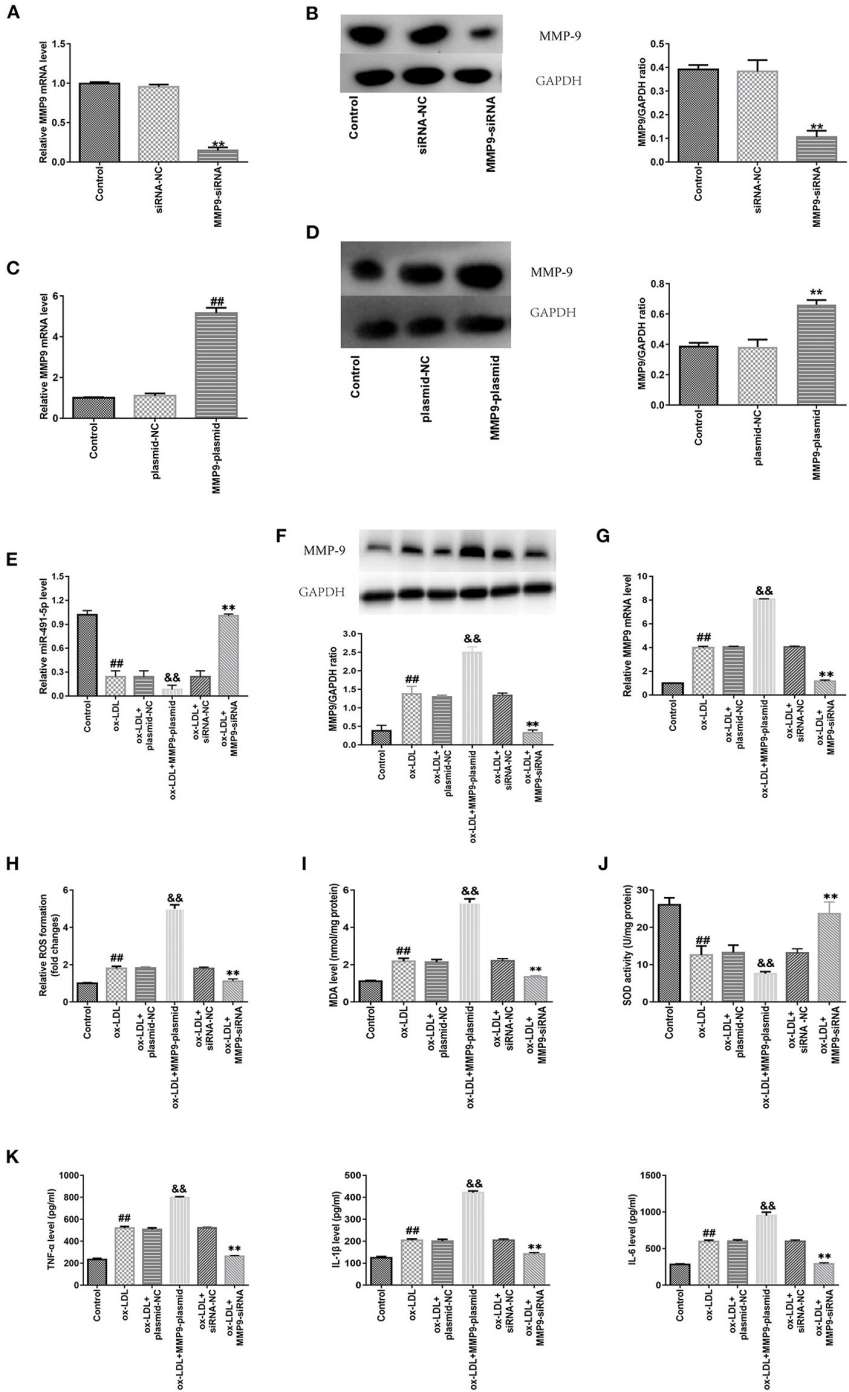
Effect of MMP-9 on the oxidative stress and inflammatory reactions in ox-LDL-induced THP-1 macrophages. **(A–D)** THP-1 macrophages were introduced with MMP-9-siRNA and MMP-9-plasmid. Transfection efficiency of MMP-9-plasmids and MMP-9-siRNA were measured with qRT-PCR, whereas MMP-9 protein was measured with western blotting. **(E–K)** Transfected THP-1 macrophages were stimulated with 50 μg/mL ox-LDL. **(E)** MiR-491-5P expression of transfected THP-1 macrophages was determined with qRT-PCR. **(F,G)** MMP-9 protein and mRNA expression of transfected THP-1 macrophages was determined by western blotting and qRT-PCR, respectively. **(H–J)** ROS and MDA levels and SOD activity were measured using commercial kits. **(K)** Levels of inflammatory factors (TNF-α, IL-1β, and IL-6) were detected using ELISA. Data are presented as the mean ± SD, *n* = 6. Compared with the control group, ^##^*P* < 0.01; compared with the plasmid-NC group, ^&&^*P* < 0.01; compared with the si-RNA-NC group, ^**^P < 0.01.

Next, protein and mRNA expression levels of MMP-9 in THP-1 macrophages were upregulated via gene introduction in the MMP-9-plasmid group and downregulated in the MMP9-siRNA group (Figures 3A–D). RT-qPCR and western blotting analyses demonstrated the ox-LDL treatment downregulated mir-491-5p (Figure 3E) and upregulated MMP-9 protein (Figure 3F) and mRNA (Figure 3G) expression levels. These changes were intensified by MMP-9-plasmid and suppressed by MMP-9-siRNA (Figures 3E–G). The MMP-9-plasmid group had higher ROS (Figure 3H) and MDA (Figure 3I) release and lower SOD activity (Figure 3J) than the ox-LDL group; however, the MMP-9siRNA group had significantly lower ROS and MDA release and higher SOD activity than the ox-LDL group (Figures 3H–J). In addition, the ox-LDL treatment markedly increased the secretory levels of TNF-α, IL-1β, and IL-6 (Figure 3K); however, these changes were aggravated by MMP-9-plasmid and inhibited by MMP9-siRNA. These results hinted that MMP-9 mediated ox-LDL-induced oxidative stress and inflammatory response in THP-1 macrophages.

### MMP-9 Overexpression Promoted Ox-LDL-Induced THP-1 Macrophage Foaming

**Figure 4:**
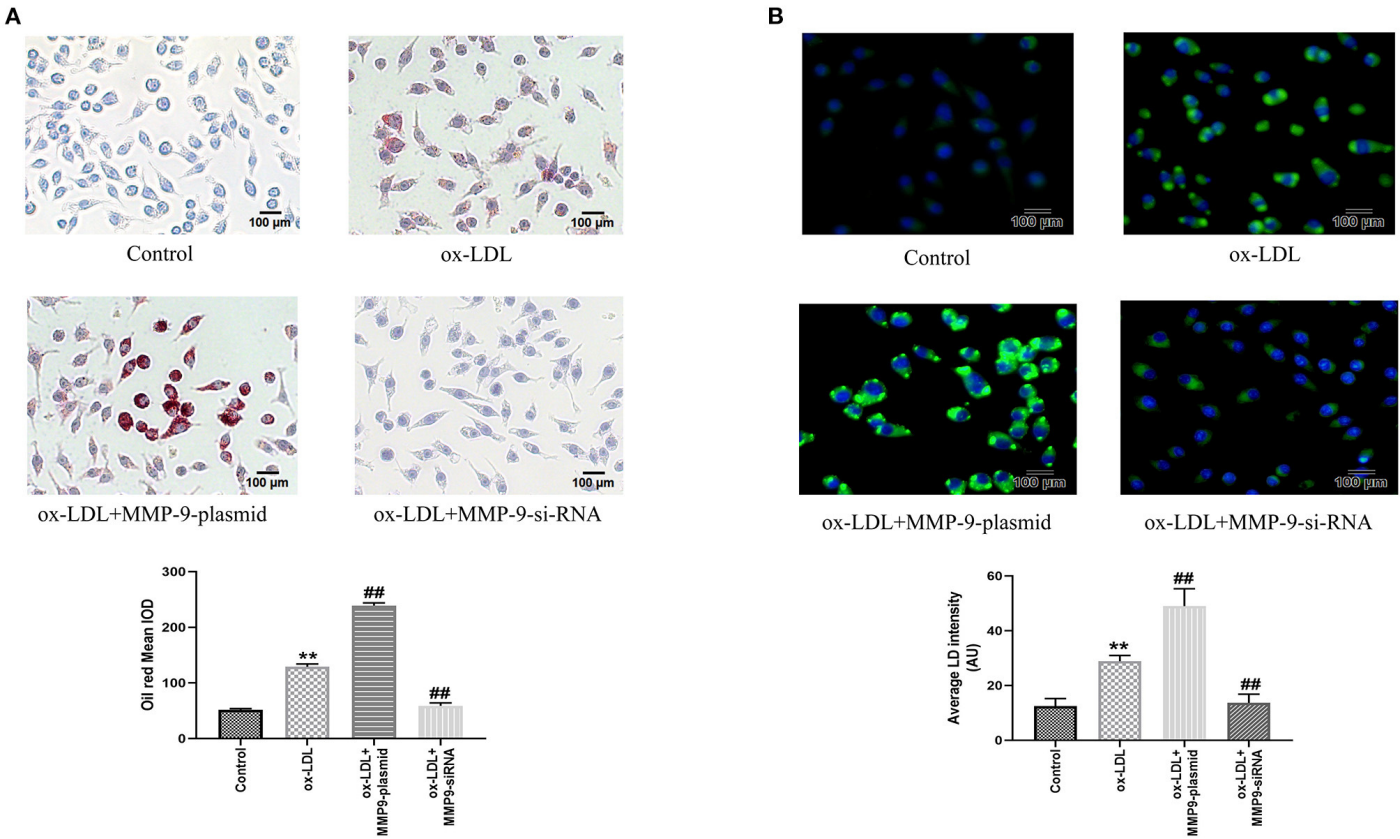
Oil Red O and BODIPY 493/503 staining after MMP-9-plasmid and MMP-9-siRNA transfections. **(A)** THP-1 macrophage cells were transfected before incubation with ox-LDL for 24 h and stained with Oil Red O (× 200, 100 μm), the intracellular lipid droplets of the mean optical density value (mean IOD) were used to reflect the degree of cellular foam. **(B)** Lipid droplets were labeled with green fluorescence and the nuclei were labeled with DAPI (blue) (× 200, 200 μm), average LD intensity was used to quantify lipid uptake. Data are presented as the mean ± SD, *n* = 6. Compared with the control group, ^**^P < 0.01; compared with the ox-LDL group, ^##^*p* < 0.01.

Meanwhile, to further investigate the effect of MMP-9 on ox-LDL-induced cell foaming, we evaluated the intracellular lipid accumulation of both MMP-9 overexpression or knockdown in THP-1 macrophages by oil red staining and fluorescence microscopy. The results showed that cytoplasmic lipid drops staining and quantitative analysis were significantly increased in the ox-LDL group, indicating THP-1 macrophage foaming (Figures 4A,B). The MMP-9-plasmid group had significantly aggravated THP-1 macrophage foaming (Figure 4A) and lipid uptake (Figure 4B) compared to the ox-LDL group, whereas MMP-9-siRNA significantly reduced ox-LDL-induced macrophage foaming and lipid uptake (Figures 4A,B). These data identified that MMP-9 not only worsened ox-LDL-induced THP-1 macrophage oxidative stress and inflammatory response but also caused cell foaming.

### MiR-491-5p Inhibited Ox-LDL-Induced THP-1 Macrophages Inflammation and Oxidative Stress by Downregulating MMP-9 Expression

**Figure 5:**
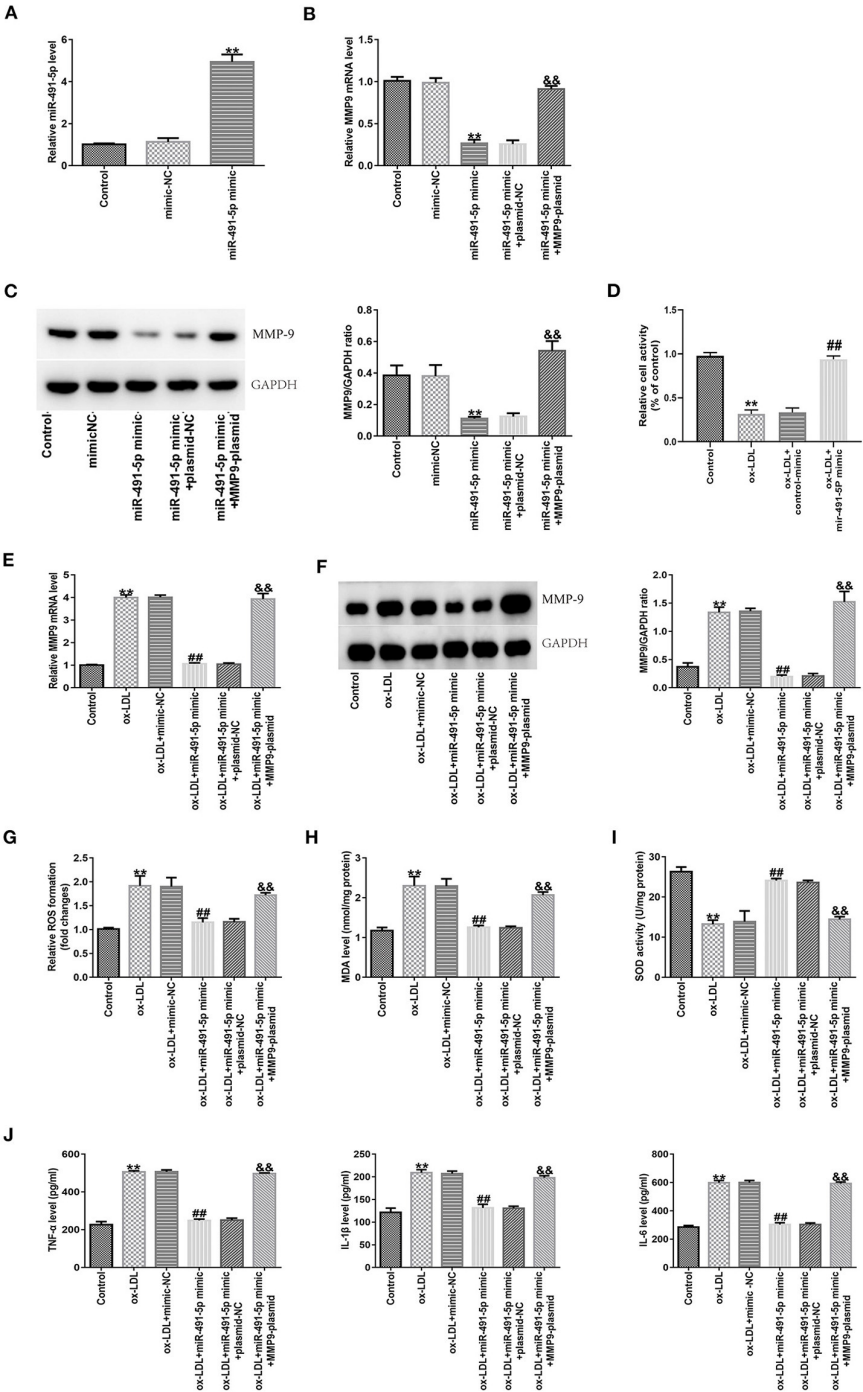
Effect of miR-491-5p and MMP-9 on ox-LDL-induced THP-1 macrophages. **(A)** The transfection efficiency of miR-491-5P mimic was determined using qRT-PCR. **(B,C)** MMP-9 mRNA and protein levels were detected in THP-1 macrophages transduced with miR-491-5P and miR-491-5P mimic+MMP-9-plasmid. **(D–J)** Transfected THP-1 macrophages were stimulated with 50 μg/mL ox-LDL. **(D)** Cell activity after mir-491-5P mimic transfection was assessed using CCK-8. **(E,F)** MMP-9 mRNA and protein were detected in ox-LDL-induced THP-1 macrophages transduced with mir-491-5p mimic and mir-491-5p mimic +MMP-9-plasmid. **(G–I)** ROS and MDA levels and SOD activity were examined using commercial kits. **(J)** Levels of inflammatory factors (TNF-α, IL-1β, and IL-6) were measured with ELISA. Data are presented as the mean ± SD, *n* = 6. Compared with the control group, ^**^P < 0.01; compared with the ox-LDL+mimic-NC group, ^##^*p* < 0.01; compared with the ox-LDL + miR-491-5Pmimic+plasmid-NC group, ^&&^*P* < 0.01.

To further demonstrate whether the effects of mir-491-5p on inflammatory cytokines and oxidative stress induced by ox-LDL by regulating MMP-9 in THP-1 macrophage cells, experiments were carried out through the transduction of mir-491-5p mimic or mir-491-5p mimic+MMP-9 plasmid into THP-1 macrophage cells before treatment with 50 μg/mL ox-LDL. After transduction of miR-491-5p into macrophages in the miR-491-5p mimic group, miR-491-5p expression was upregulated (Figure 5A), and mRNA (Figure 5B) and protein (Figure 5C) expression levels of MMP-9 in THP-1 macrophages were significantly downregulated. These changes were reversed after co-transfection with the MMP9-plasmid in the miR-491-5p mimic+ MMP-9-plasmid group (Figures 5B,C). To investigate the role of mir-491-5P in ox-LDL-mediated damage, THP-1 macrophages transduced with mir-491-5p mimic or mimic-NC were stimulated with 50 μg/mL ox-LDL. CCK-8 analysis showed that high mir-491-5p expression increased the viability of THP-1 macrophages compared to the ox-LDL treatment alone (Figure 5D). MMP-9 mRNA (Figure 5E), protein (Figure 5F), ROS, MDA (Figures 5G,H), IL-6, IL-1β, and TNF-α (Figure 5J) expression levels were downregulated, and SOD activity was improved (Figure 5I) in the miR-491-5P mimic group compared to the ox-LDL group. In contrast, these observational indicators were reversed in the miR-491-5P mimic+MMP-9-plasmid group (Figures 5E–J). These results suggested that mir-491-5p inhibited ox-LDL-induced oxidative stress and inflammatory cytokines in THP-1 macrophages by inhibiting MMP-9 expression.

#### Mir-491-5p Attenuated Ox-LDL-Induced THP-1 Macrophages Foaming

**Figure 6:**
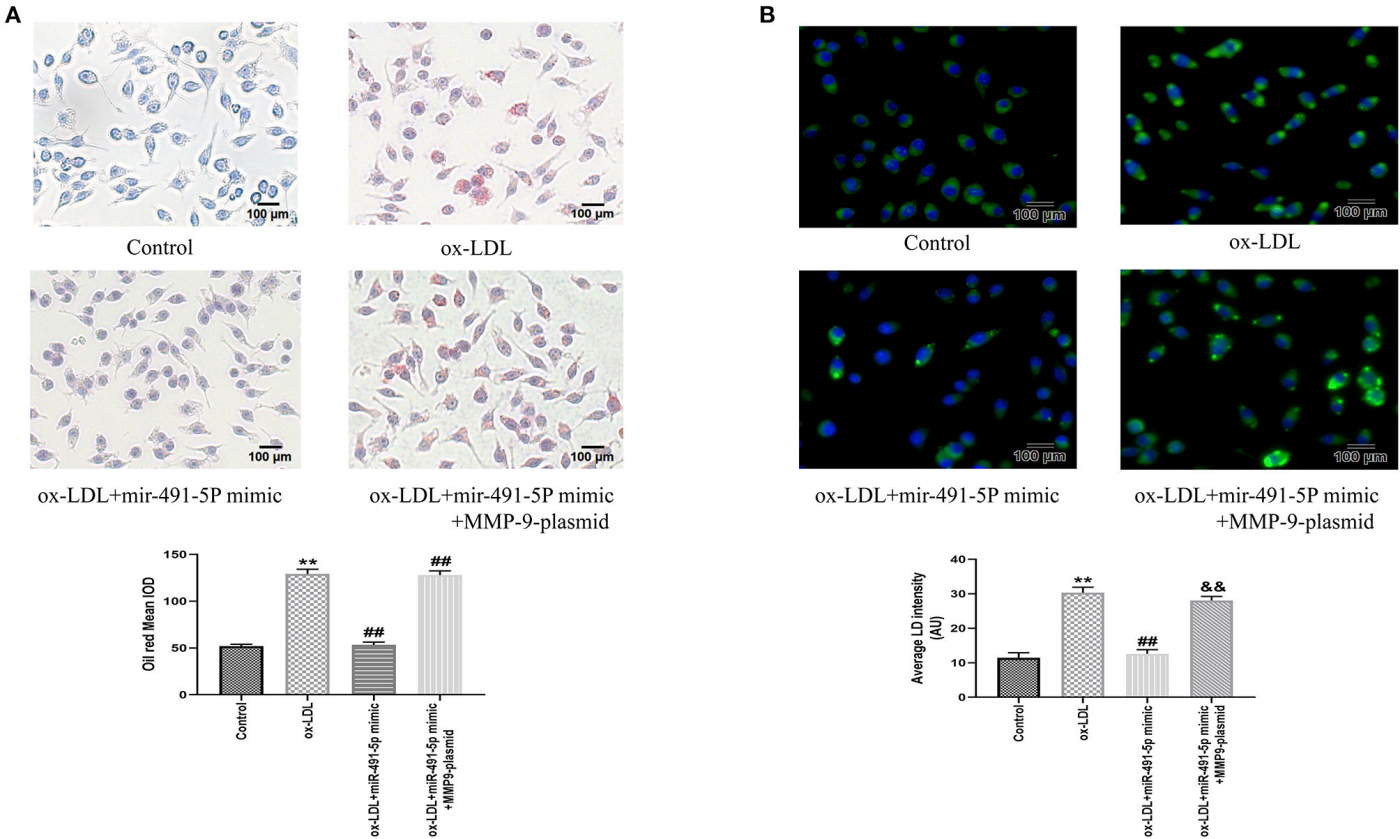
Oil Red O and BODIPY 493/503 staining after mir-491-5P mimic and mir-491-5P mimic +MMP-9-plasmid transfections. **(A)** THP-1 macrophage cells were transfected before incubation with ox-LDL and staining with Oil Red O (× 200, 100 μm), the intracellular lipid droplets of the mean optical density value (mean IOD) were used to reflect the degree of cellular foam. **(B)** Lipid droplets were labeled with green fluorescence and the nuclei were labeled with DAPI (blue) (× 200, 100 μm), average LD intensity was used to quantify the lipid uptake. Data are presented as the mean ± SD, n = 6. Compared with the control group, ^**^P < 0.01; compared with the ox-LDL group, ^##^*p* < 0.01; compared with the ox-LDL+mir-491-5pmimic group, &&*p* < 0.01.

Similarly, we observed the effect of mir-491-5p on ox-LDL-induced THP-1 macrophage foaming by oil red staining and fluorescence microscopy. Oil Red O and BODIPY 493/503 staining showed that ox-LDL induced the THP-1 macrophage foaming and increased content lipid droplets contentment in cells (Figures 6A,B). The mir-491-5P mimic group had significantly lower THP-1 macrophage foaming (Figure 6A) and lipid uptake (Figure 6B) than the ox-LDL group, whereas the mir-491-5P mimic+MMP-9-plasmid significantly reversed these phenomena (Figures 6A,B). These results suggested that miR-491-5p inhibited ox-LDL-induced THP-1 macrophage foaming by inhibiting MMP-9 expression.

## Discussion

LDL is the primary lipoprotein inducing AS, and ox-LDL cannot be easily recognized and scavenged by the relevant receptors (3). The macrophages differentiated by monocytes can phagocytize ox-LDL to form lipid-laden foam cells, thus becoming a component of plaques (4, 5). Macrophage foaming and increased intracellular lipid content can be used as markers of the severity of arteriosclerosis caused by lipids. Macrophage foaming and lipid accumulation were visual after Oil Red O and BODIPY 493/503 staining, and the intracellular lipid droplets were quantitatively assessed using the mean optical density value (mean IOD) and average LD intensity, respectively. Ox-LDL can stimulate macrophages to release oxygen free radicals and inflammatory factors, resulting in local tissue necrosis and triggering and facilitating the formation of plaques (21). ROS and MDA levels reflect the lipid peroxidation degree, and SOD is an antioxidant factor (2). Upregulated expression of TNF-α, IL-1β, and IL-6 infer aggravation of the inflammatory response (12). Further, ox-LDL can stimulate macrophages to release MMP-9 and elevate MMP-9 expression level in plaques (22, 23). In the present study, after the macrophages were co-incubated with ox-LDL, the MMP-9, ROS, MDA, TNF-α, IL-1β, and IL-6 expression levels were upregulated in the model group. Simultaneously, SOD activity was reduced, and foam cell formation and lipid accumulation were increased, validating the expected effect of ox-LDL on the macrophages, consistent with previous studies (19, 24). In contrast, the miR-491-5P expression level was lowered. Further investigations illustrated that the effects of ox-LDL on macrophages were lessened in the ox-LDL + miR-491-5P mimic group after the miR-491-5P expression level was elevated via gene introduction. This phenomenon was nullified in the ox-LDL + miR-491-5Pmimic+MMP-9plasmid group with high miR-491-5P and MMP-9 expression levels. However, the MMP-9 expression level increased in the MMP-9-plasmid group, and the corresponding oxidative stress and inflammatory responses and the degree of cell foaming were enhanced. Those in the MMP-9-siRNA group with MMP-9 silencing showed the opposite trend, indicating that ox-LDL might aggravate the oxidative stress and inflammatory responses of THP-1 macrophages via the repression of the miR-491-5P/MMP-9 axis.

Recently, extensive research attention has focused on the biological functions of miRNA. MiR-491-5P, a type of miRNA, can regulate the expression of the MMP-9 gene by binding to the 3′UTR non-coding site of MMP-9 (25, 26). Recent *in vitro* studies have shown that miR-491-5P can inhibit the growth and migration of vascular smooth muscle cells through the targeted inhibition of MMP-9 expression (17). Furthermore, clinical studies have indicated that the binding site for miR-491-5P and MMP-9 undergoes mutation due to the gene polymorphism of MMP-9; therefore, miR-491-5P fails to bind to MMP-9, the *in vivo* MMP-9 expression level of the patient is increased, and the risk of atherosclerotic cerebral infarction is amplified (18). According to the present study, miR-491-5P repressed the ox-LDL-induced oxidative stress and inflammatory responses of THP-1 macrophages through the targeted inhibition of MMP-9, and cell foaming and lipid uptake were significantly decreased, further demonstrating the role of the miR-491-5P/MMP-9 pathway in AS attack.

However, the upstream promoter of miR-491-5P must be further clarified. Transcription factors, epigenetic modification, and the conversion of pri-miR to miRNA might play essential roles in regulating miRNA expression. Previous tumor cell studies found that the Foxi1 protein, belonging to the forkhead gene family, is a potential upstream regulator of miR-491-5p, and can bind to miR-491-5p binding sites to regulate the expression of miR-491-5p (27). Another study found that circ0001361, a circular RNA member, can specifically inhibit miR-491-5P (28) and upregulate MMP-9 to promote cell proliferation and migration. Although the existing literature indicates that the Foxi1 protein and circ0001361 are upstream of miR-491-5p, it is unclear whether the Foxi1 protein or CIRC0001361 play a role in mediating the ox-LDL-induced oxidative stress and inflammatory responses of THP-1 macrophages. If so, it remains unclear what their upstream factors are. The pathway between ox-LDL and miR-491-5p appears to be complex and requires further exploration in future studies.

As a proteolytic enzyme, MMP-9 degrades the fibers in plaque ECM. The plaques become soft, fiber caps become thin, and the plaques become unstable, increasing the probability of clinical cardiovascular events (7). Another study has shown that MMP-9 exerts a “positive feedback” regulatory effect on inflammatory factors, and its high expression can aggravate the inflammatory response (9). IL-1β and IL-8 are essential inflammatory cytokines; MMP-9 facilitates the transformation of the inactive precursor IL-1β into active IL-1β by activating the IL-1β invertase (10) and pyrolyzes IL-8 to enhance its chemotactic activity and further aggravate the inflammatory response (11). In inflammatory diseases, MMP-9 gene silencing can also restrict ROS accumulation (29), obstruct MMP-9 activation, repress the redox reaction, and relieve neuroinflammation (30). The present study showed that high MMP-9 expression further enhanced the oxidative stress and inflammatory responses to ox-LDL. MMP-9 silencing led to the opposite outcome, further verifying the oxidative stress-promoting and pro-inflammatory effects of MMP-9. Moreover, the gene introduction downregulated the miR-491-5P expression level under high MMP-9 expression. In the MMP-9 gene silencing condition, the miR-491-5P level was upregulated, suggesting that MMP-9 might exert negative feedback on miR-491-5P. Nevertheless, the mechanism remains unclear.

Several limitations need to be considered in the present study. The *in vitro* experiment confirmed that miR-491-5P/MMP-9 mediated a possible induction mechanism of ox-LDL in AS. However, as the formation of arterial plaques could not be directly observed, this requires further verification through follow-up *in vivo* and pathophysiological studies of plaque formation. In addition, the specific mechanism underlying MMP-9 regulation by ox-LDL remains unclear.

In summary, ox-LDL was found to aggravate the oxidative stress and inflammatory reactions of THP-1 macrophages by downregulating the miR-491-5P expression level and reducing the inhibiting effect of miR-491-5P on MMP-9.

## Data Availability Statement

The original contributions presented in the study are included in the article/supplementary material, further inquiries can be directed to the corresponding author.

## Author Contributions

YL contributed to the conception of the study, contributed significantly to analysis and manuscript preparation, and performed the data analyses and wrote the manuscript. YL and EZ were responsible for the experiment. EZ helped perform the analysis with constructive discussions. WZ was responsible for the review and revision of the paper and supported the financial acquisition for the project leading to this publication. All authors contributed to the article and approved the submitted version.

## Conflict of Interest

The authors declare that the research was conducted in the absence of any commercial or financial relationships that could be construed as a potential conflict of interest.

## Publisher's Note

All claims expressed in this article are solely those of the authors and do not necessarily represent those of their affiliated organizations, or those of the publisher, the editors and the reviewers. Any product that may be evaluated in this article, or claim that may be made by its manufacturer, is not guaranteed or endorsed by the publisher.
